# Associations between oral hormonal contraceptives and internalising problems in adolescent girls

**DOI:** 10.1192/bjo.2024.859

**Published:** 2025-03-04

**Authors:** Nadie H. M. Bosmans, Milan Zarchev, Leonie Berges, Astrid M. Kamperman, Eline M. P. Poels, Witte J. G. Hoogendijk, Nina H. Grootendorst-van Mil

**Affiliations:** Department of Psychiatry, Erasmus MC University Medical Center, Rotterdam, The Netherlands; Epidemiological and Social Psychiatric Research Institute, Department of Psychiatry, Erasmus MC University Medical Center, Rotterdam, The Netherlands

**Keywords:** Adolescent, internalising problems, oral contraceptives, population-based cohort

## Abstract

**Background:**

Oral contraceptive pills (OCP) have received increased critical attention recently owing to their perceived link with mental health, especially among adolescent girls. The empirical literature, however, includes mixed findings on whether OCP use is associated with poorer mental health.

**Aims:**

To examine the association between the use of OCP and internalising problems in adolescent girls.

**Methods:**

This study was embedded in the iBerry study, a population-based cohort of adolescents oversampled for behavioural and emotional problems from the greater Rotterdam area, The Netherlands. In 372 girls, internalising problems were measured using the Youth Self Report, and use of OCP was determined by parental interview and self-report questionnaire across two subsequent waves (mean ages 14.9 and 17.9 years, respectively). Multiple regression analyses were performed to determine the association. Analyses were adjusted for various sociodemographic factors and adjusted for previous internalising problems assessed at a mean age of 14.9 years.

**Results:**

In total, 204 girls (54.8%) used OCP. OCP use was associated with fewer internalising problems in adolescent girls compared with non-use (adjusted β = −2.22, 95% CI [−4.24, −0.20]; *P* = 0.031).

**Conclusions:**

In this research, we found that adolescent girls using OCP reported fewer internalising problems compared with non-users. This association was most prominent for girls with pre-existing internalising problems. Although healthy user bias may have a role, our observations suggest a potential therapeutic benefit for those with greater baseline challenges.

Mental disorders, notably depression and anxiety, are leading contributors to the global non-fatal burden of disease, peaking in adolescence to middle age.^1^ Previous research has demonstrated sex-related differences in psychopathology, with women more prone to internalising disorders.^2,3^ This increased risk has been ascribed partly to changes in endocrine control of the reproductive system in women.^2^ Oestrogen fluctuations, particularly during hormone-sensitive transitional periods, are related to women’s heightened depression risk.^2,3^ It is hypothesised that hormonal contraceptives may alter oestrogen levels, inducing fluctuations that could potentially precipitate depressive symptoms.^4,5^ This suggests that adolescent girls using such contraceptives may have heightened risks of internalising problems.

In recent years, there has been an increase in critical thinking regarding mood-related side-effects of hormonal contraceptives, fuelled by social media discussions.^6^ In Europe and North America, oral contraceptive pills (OCP) represented the most popular method of contraception in 2019, used by 17.8% of women.^7^ Notably, the use of OCP among Dutch women aged 18–49 declined by 6% over the past 6 years.^8^ A significant factor behind this shift is that one-third of women not using contraceptives report avoiding hormones.^8^ Mood changes are a primary concern, leading many to discontinue or switch their contraceptive methods.^9^ Women often cite hormone-related apprehensions and a fear of psychological side-effects, compounded by a perceived lack of comprehensive counselling on these risks.^9^ This evolving perspective, including concerns over psychological side-effects, reflects a wider caution towards hormonal contraceptives.

The apprehension towards hormones and the perceived insufficiency of counselling might stem from the ambiguous evidence regarding the impact of hormonal contraceptives on mental health. A large nationwide study in Denmark found an association between hormonal contraceptive use and both antidepressant use and initial depression diagnosis.^10^ This association was even higher for adolescents, suggesting a potential age-specific side-effect.^10^ Reinforcing this concern, a recent cohort study found that the use of oral contraceptives during adolescence might increase the risk of depression later in life.^11^ Another study found an association between hormonal contraceptives and reporting more depressive symptoms, only for girls aged 16 years; from that age upwards there was no association found.^12^ These findings hint at a potential heightened sensitivity to depression risks among adolescent first-time users of hormonal contraceptives.

A review based on observational studies and randomised controlled trials, however, concluded that there was no consistent evidence for negative effects of hormonal contraceptives in the general population.^13^ Two large epidemiological studies found no increased risk of depressive disorders in adolescents and young women using oral hormonal contraceptives.^14,15^ A systematic review of randomised controlled trials also found no increase in depressive symptoms with hormonal contraceptives compared with placebo, although the authors noted a lack of data on first-time adolescent users.^16^ This gap in research is critical, as adolescents represent a demographic of interest considering the peak in burden of disease. By contrast, two national studies concluded that hormonal contraceptives were associated with better mood and reduced levels of depressive symptoms.^17,18^ This demonstrates the ambiguous evidence regarding hormonal contraceptive use and internalising problems.

Given the substantial burden of internalising problems and the growing concern regarding psychological side-effects of oral contraceptives, combined with existing research ambiguities, there is need for a better understanding of this issue. This study was designed to explore the relationship between the use of hormonal contraceptives and the occurrence of internalising problems, such as depression and anxiety, in adolescent girls. We specifically aimed to address a gap in existing research by carefully considering any internalising problems that the individual might have experienced before they began using contraceptives. In addition, our follow-up analysis investigated secondary outcomes, including withdrawn–depressive and anxious–depressive problems, as well as defining cut-off points for clinical and borderline-clinical scores on internalising problems. We hypothesised that the use of OCPs would be associated with an increase in internalising problems among adolescent girls.

## Methods

### Study design and participant subsection

The current study is embedded in the iBerry (Investigating Emotional and Behavioral Risk in Rotterdam Youth) study, performed at the Department of Psychiatry of the Erasmus MC University Medical Center in Rotterdam, The Netherlands. The iBerry study is a prospective longitudinal cohort study of adolescents oversampled for risk of developing psychopathology, conducted in the greater Rotterdam region of The Netherlands. The overall goal of the study is to examine the transition from non-specific psychiatric symptoms in adolescence to psychiatric disorders later in life. The study design and cohort profile have beene described elsewhere in detail.^19,20^ Adolescents were selected by using a Dutch translation of the Strengths and Difficulties Questionnaire-youth (SDQ-Y), a screening self-report questionnaire that measures emotional and behavioural problems.^21^ Adolescents with a score in the top 15% on the SDQ-Y were oversampled at a 2.5:1 ratio to adolescents with a lower score. Participants were enrolled between September 2015 and September 2019. At baseline, a total of 1022 adolescents (boys and girls) with a mean age of 15 years were enrolled (51.1% girls). Participants, together with a parent, were invited to the research centre of the iBerry study. Before the visit, participants received information about the study and signed an informed consent form. A research employee, blind to the adolescents’ SDQ-Y status, performed multiple interviews, questionnaires, neuropsychological tests and biological measurements to assess determinants of psychopathology. After completing the visit, participants received an incentive. Participants are invited every 2–3 years for follow-up measurements. For the current study, we used data on girls included in the first follow-up measurement (mean age 17.9 years). Our adjusted analyses also incorporate information on contraceptive use and internalising problems collected at baseline (mean age 14.9 years). Data about the use of OCP were missing for 59 of the 431 girls in total, leaving 372 girls (86.3%) in the sample for analyses.

The authors assert that all procedures contributing to this work comply with the ethical standards of the relevant national and institutional committees on human experimentation and with the Helsinki Declaration of 1975, as revised in 2013. All procedures involving human subjects and/or patients were approved by the Medical Ethics Review Committee of the Erasmus MC University Medical Center (MEC-2015-007, MEC-2018-1472).

### Measures

#### OCP use

Data on OCP use was obtained at baseline (age 15 years) and at the first follow-up visit (age 18 years) via two instruments. First, the Trimbos/iMTA Questionnaire for Costs associated with Psychiatric Illness (TIC-P),^22^ an interview for the parent, was used to ask about the use of medication prescribed by a doctor for the adolescent. The parent was asked whether their child had used any medication in the past 2 years. The available response options were ‘yes’ and ‘no’. When the parent responded with ‘yes’, the medication type was noted. The TIC-P interview was also conducted at age 15 years to assess prescribed medication in an identical manner, except that lifetime use up to age 15 years was inquired about. This allowed us to assess the history of prior contraceptive use before the measurement at age 18 years.

Second, in a questionnaire on sexuality at age 18 years, participants were asked the question ‘Have you ever had sexual intercourse?’. If the response was ‘yes’, a subsequent question on use of contraceptives followed: ‘Do you or your partner use contraception?’. The available response options were ‘yes, always’, ‘sometimes’, ‘no, never’ or ‘I don’t remember’. When participants chose one of the initial two response options, they were subsequently asked a follow-up question regarding the specific contraception used and its frequency.

The responses obtained at age 18 years from the TIC-P interview and sexuality questionnaire were combined to evaluate the use of OCP as the main predictor. The participant was classified as an OCP user when OCP use was documented using either of the aforementioned measurements.

#### Internalising problems

Internalising problems were assessed at baseline for adjustment purposes and at the first follow-up visit to measure outcomes. This was done using a Dutch version of the Youth Self-Report (YSR) questionnaire from the Achenbach System of Empirically Based Assessment.^23^ This self-report questionnaire for adolescents aged 11 to 18 years consisted of 112 statements regarding skills and emotional and behavioural problems during the past 6 months. The possible response options to the statements were 0 (not at all), 1 (a bit or sometimes) or 2 (clearly or often). The subscale for internalising problems contained 31 statements about withdrawn–depressive problems, as well as anxious–depressive problems and somatic complaints. Sum scores for the subscale internalising problems were calculated, ranging from 0 to 62. Higher scores were indicative of more problems. The sum scores were used for assessment of the main outcome. A maximum of 25% missing data was accepted for the YSR. If there were any items missing on a subscale, the score was multiplied by the total number of items per scale divided by the number of filled-in items. An additional scoring metric was devised to categorise the adolescents’ sex- and age-specific scores by determining whether they fell within the borderline clinical range (exceeding the 93rd percentile) or the clinical range (surpassing the 98th percentile). The reliability and validity of this Dutch version of the questionnaire was good.^24^ Cronbach’s alpha for the YSR questionnaire was 0.91.

#### Covariates

Potential confounders were obtained from the previous literature.^10–12,14–18^ Age and ethnic background were obtained from self-report questionnaires. Non-verbal IQ score was assessed using two subtest of the Snijden Oomen Non-verbal Intelligence Test – Revised, which is correlated strongly with total IQ.^25^ Monthly net household income, as a proxy for socioeconomic status, was obtained via parental questionnaire and was divided into four groups: ≤1599, 1600–2399, 2400–4399 and ≥4400 euros. Ethnic background was based on parental birth country and categorised into two groups: Dutch and non-Dutch.

### Statistical analysis

A linear regression analyses was conducted to examine the association between OCP use and internalising problems, taking into account potential sociodemographic confounders. Subsequent analyses were conducted, adjusting for internalising problems at baseline, OCP use at baseline and sexual activity. We report unstandardised beta coefficients and the corresponding 95% confidence intervals. As measure of effect size, Cohen’s *d* is reported. Assumptions for linear regression were checked. All analyses were performed in R (version 4.2.3).^26^

Several follow-up analyses were conducted. First, the internalising problems outcome was substituted with the withdrawn–depressive problem score from the YSR. Second, the outcome was replaced with the anxious–depressive problem score. The same covariates as those used in the fully adjusted model above were included in the analysis, and the same estimates were reported. Third, the continuous internalising problems outcome was transformed into a binary cut-off categorical variable. The cut-offs reflected whether adolescents scored in the normal internalising problems range or in the borderline clinical range. Fourth, a binary cut-off variable was used for the outcome, this time indicating whether adolescents scored in the normal–borderline range versus the clinical range. For the latter two analyses, a logistic regression was used to model the binary outcomes. The estimates reported were odds ratios and the corresponding 95% confidence intervals. As a measure of effect size, the marginal risk difference between those who took and did not take OCP was reported. Fifth, to further address healthy user bias, girls who had discontinued OCP use at baseline (*n* = 8) were included as OCP users, and the associations were re-estimated as in the primary analysis. Sixth, we excluded OCP users who had started use at baseline and continued at the first follow-up (*n* = 36). The purpose of this was to remove people who persistently used OCP and therefore might have been particularly satisfied with its effects on mental health. Finally, we re-ran the primary analysis but added an interaction term between OCP use and prior mental health problems. We used estimated marginal means and prediction plots to investigate whether the association with OCP was different among girls with pre-existing mental health problems.

Thirty-two (8.7%) adolescents had missing data for monthly household income, 21 (5.7%) for history of sexual activity, 18 (4.9%) for baseline OCP use, 16 (4.3%) for ethnic background and eight (2.2%) for baseline internalising problems. The problem of missing data was handled using multiple imputation as implemented in R package ‘mice’.^27^

### Ethics statement

The authors assert that all procedures contributing to this work comply with the ethical standards of the relevant national and institutional committees on human experimentation and with the Helsinki Declaration of 1975, as revised in 2013. All procedures involving human subjects/patients were approved by the Medical Ethics Review Committee of the Erasmus MC University Medical Center (MEC-2015-007, MEC-2018-1472). Both adolescents and their parent(s) or legal guardians gave written informed consent to participate in the study.

## Results

Descriptive statistics of OCP users and non-users are shown in Table 1. The mean age of the adolescent girls was 14.9 years at baseline (s.d. = 0.9) and 17.9 years (s.d. = 0.8) at follow-up; in total, 293 (78.8%) were of Dutch origin, comprising 83.1% of the OCP users and 81.4% of the non-users. The average household income was mostly moderate-high (2400–4399 euros monthly). Overall, 204 (54.8%) adolescent girls had used OCP at some time during the past 2 years. Of those not using OCP (169; 45.2%) at the follow-up measurement, eight girls (5.0%) had discontinued use after the baseline measurement. OCP users were more likely to be sexually active (77.3%) compared with non-users (30.0%).

**Table 1:** Characteristics of study participants (*n* = 372) stratified by oral contraceptive pill (OCP) use^a^

	OCP users *n* = 204	OCP non-users *n* = 168	*P*-value^b^
Age, years			0.23
	18.0 (0.7)	17.9 (0.8)	
Ethnic background			0.75
Dutch	162 (83.1%)	131 (81.4%)	
Non-Dutch	33 (16.9%)	30 (18.6%)	
Urbanicity of living environment			0.32
Rural	48 (23.5%)	44 (26.2%)	
Suburban	42 (20.6%)	24 (14.3%)	
Urban	114 (55.9%)	100 (59.5%)	
Educational level			0.21
Special-needs secondary education	3 (1.5%)	5 (3.0%)	
Pre-vocational secondary education	98 (49.7%)	58 (35.2%)	
Higher general secondary education	50 (25.4%)	43 (26.1%)	
Pre-university educational level	38 (19.3%)	41 (24.8%)	
Combined educational level	8 (4.1%)	18 (10.9%)	
Monthly net household income, euros			0.06
<1599	23 (12.4%)	10 (6.5%)	
1600–2399	28 (15.1%)	19 (12.3%)	
2400–4399	101 (54.6%)	82 (53.2%)	
>4400	33 (17.8%)	43 (27.9%)	
IQ score^c^	100.3 (12.9)	100.8 (13.6)	0.71
Sexually active, yes	153 (77.3%)	45 (30.0%)	<0.001
Previous OCP use^c^	36 (18.5%)	8 (5.0%)	<0.001

a. Data are presented as *n* (%) or mean (s.d.).

b. Welch two-sample *t*-test; Fisher’s exact test.

c. Measured at age 15.

### Association between OCP use and internalising problems

OCP use during the past 2 years was associated with lower mean scores for internalising problems, adjusting for age (unstandardised β coefficient −2.27; 95% CI [–4.27, –0.28]; *P* = 0.026). Results of linear regression analyses are shown in Table 2. After correction for sociodemographic variables in the second model, OCP use was associated with lower adjusted mean scores for internalising problems (β = −2.92; 95% CI [−4.95, −0.88]; *P* = 0.005). After full adjustment in the third model, OCP use was associated with lower adjusted mean scores for internalising problems (β = −2.22; 95% CI [−4.24, −0.20]; *P* = 0.031). Thus, adolescent girls who had used OCP at some point during the past 2 years, on average, scored 2.22 points lower on the YSR internalising problems scale for the past 6 months, in comparison with those who had not used OCP during the past 2 years, and the effect size was small (Cohen’s *d* = −0.23 95% CI [−0.44, −0.03]).

**Table 2:** Associations between oral contraceptive pill (OCP) use and internalising problems in adolescent girls

	Unstandardised β coefficient	95% CI		*P*-value	Cohen’s *d*	95% CI	
		Lower limit	Upper limit			Lower limit	Upper limit
Internalising problems, adjusted for:							
Age	−2.27	−4.27	−0.28	0.026	−0.23	−0.44	−0.03
Age and sociodemographic variables^a^	−2.92	−4.95	−0.88	0.005	−0.39	−0.66	−0.11
Age, sociodemographic variables,^a^ prior internalising problems,^b^ previous OCP use and sexual activity (fully adjusted)	−2.22	−4.24	−0.20	0.031	−0.23	−0.44	−0.02

a. Sociodemographic variables include IQ score, ethnic background and monthly net household income.

b. Prior internalising problems evaluated at baseline.

To answer the secondary outcome questions, linear regression analyses were performed, replacing internalising problems with withdrawn–depressive problems and anxious–depressive problems. After full adjustment, OCP use was associated with lower adjusted mean scores for withdrawn–depressive problems (β = −0.87; 95% CI [−1.52, −0.23]; *P* = 0.008), with a small effect size (Cohen’s *d* = −0.36; 95% CI [−0.62, −0.09]). OCP use was also associated with lower adjusted mean scores for anxious–depressive problems (β = −1.49; 95% CI [−2.60, −0.38]; *P* = 0.009), with a small effect size (Cohen’s *d* = −0.34; 95% CI [−0.59, −0.09]). Results are shown in Table 3.

**Table 3:** Associations between oral contraceptive pill (OCP) use and withdrawn–depressive and anxious–depressive problems in adolescent girls

	Unstandardised β coefficient	95% CI		*P*-value	Cohen’s *d*	95% CI	
		Lower limit	Upper limit			Lower limit	Upper limit
Withdrawn–depressive problems, score, fully adjusted^a^	−0.87	−1.52	−0.23	0.008	−0.36	−0.62	−0.09
Anxious–depressive problems, score, fully-adjusted^a^	−1.49	−2.60	−0.38	0.009	−0.34	−0.59	−0.09

a. Adjusted for age, IQ score, ethnic background, monthly net household income, prior internalising problems, previous OCP use and sexual activity.

Logistic regression analyses were performed for the association between OCP use and the cut-offs for clinical and borderline-clinical range for internalising problems. Results are shown in Table 4. The association between OCP use and internalising problems was significant in the same direction when comparing adolescents in the normal and borderline range with those in the clinical range (odds ratio 0.43; 95% CI [0.22, 0.81], *P* = 0.009). There were 12.3% more girls with clinical internalising problems among those who did not use OCP (risk difference −12.3%; 95% CI [−21.5%, −3.1%]). However, there was a non-significant difference between adolescents in the normal range and those in the borderline and clinical range combined (risk difference 0.70; 95% CI [0.40, 1.22], *P* = 0.208).

**Table 4:** Associations between oral contraceptive pill (OCP) use and internalising problems

	Odds ratio	95% CI		*P*-value	Risk difference (%)	95% CI	
		Lower limit	Upper limit			Lower limit	Upper limit
Borderline and clinical internalising problems combined, fully adjusted^a^	0.70	0.40	1.22	0.208	−7.1	−18.1	3.9
Clinical internalising problems, fully adjusted^a^	0.43	0.22	0.81	0.009	−12.3	−21.5	−3.1

a. Adjusted for age, IQ score, ethnic background, monthly net household income, prior internalising problems, previous OCP use and sexual activity.

**Fig. 1:**
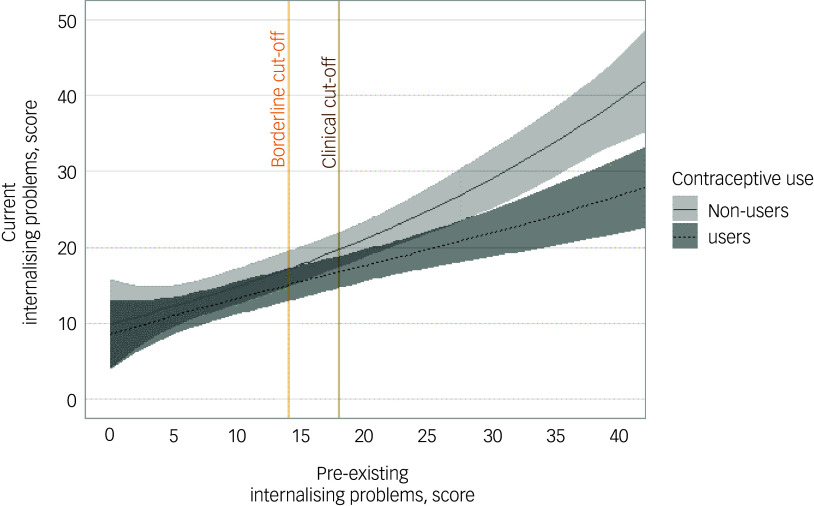
Interaction plot for oral contraceptive pill (OCP) users and non-users as a function of pre-existing internalising problems. All predictions were adjusted for age, IQ score, ethnic background, monthly net household income, prior internalising problems, previous OCP use and sexual activity.

Further, including girls who used OCP at baseline but discontinued it later (*n* = 8) as OCP users did not change the association (unstandardised β coefficient −3.28; 95% CI [−5.25, −1.30]; *P* < 0.001). Likewise, excluding participants who used OCP at both baseline and first follow-up measurement (*n* = 36) did not affect the association (β −2.69; 95% CI [−4.72, −0.66]; *P* = 0.010). In a final sensitivity analysis, we found a significant interaction between OCP use and prior internalising problems at baseline (*P* = 0.009). Estimated marginal means indicated that the association between OCP and current internalising problems was not significant among those with no pre-existing internalising problems (unstandardised β coefficient 1.26; 95% CI [−4.37, 1.84]; *P* = 0.425) or those with borderline pre-existing internalising problems (unstandardised β coefficient −2.08; 95% CI [−4.63, 0.47]; *P* = 0.110). For girls above the clinical cut-off for pre-existing internalising problems, the association was significantly negative (unstandardised β coefficient −2.94; 95% CI [−5.44, −0.44]; *P* = 0.021). The estimated predictions for OCP users and non-users as functions of pre-existing internalising problems are presented in Fig. 1. The risk for current internalising problems became higher for non-users about half-way between the borderline and clinical cut-offs for pre-existing internalising problems.

## Discussion

The current study, embedded within a population-based cohort of adolescents, explored the relationship between the use of OCP and internalising problems in adolescent girls. Our findings suggest that girls who had used OCP experienced fewer internalising problems relative to their non-using counterparts. The negative association was observed consistently across both withdrawn–depressive and anxious–depressive problem categories. This association between OCP use and fewer internalising problems was most prominent for girls with pre-existent internalising problems. Our findings were contrary to our initial hypothesis and diverged from prior research indicating negative effects of oral hormonal contraceptives on adolescent mental health.^10,12^ They were also contrary to the findings of a series of studies reporting no significant differences in depressive symptoms when comparing users and non-users of oral hormonal contraceptives.^13–16^

Our findings align with those of multiple studies that suggested associations of hormonal contraceptives with reduced mood and depressive symptoms.^17,18^ Specifically, a 2012 national survey in Finland indicated that current oral contraceptive use was linked to improved mood, with a modest effect size.^17^ Furthermore, a US study conducted in 2013 among sexually active young women (aged 25–34 years) found that hormonal contraceptives potentially decreased depressive symptoms.^18^

The differences in our findings compared with those of previous studies could have been due to serval factors, including the characteristics of our population, which was oversampled for high-risk individuals. We identified a significant interaction between OCP use and baseline internalising problems, indicating that the association was most protective among girls with prior psychopathology and non-significant in girls with no prior psychopathology. This pattern aligns with the findings of a recent systematic review, which reported a protective association between OCP and depression but only in samples with pre-existing problems.^28^ Ours is the first study to quantify the contribution of pre-existing internalising problems within a single sample. One potential explanation for this pattern is that OCP are frequently prescribed to treat conditions with hormonal psychopathology such as acne, polycystic ovary syndrome or premenstrual syndrome.^29,30^ Given that our high-risk sample may simultaneously have been enriched with girls suffering from polycystic ovary syndrome or premenstrual syndrome, the observed protective effect on internalising symptoms may reflect the therapeutic benefits of OCP in these subgroups.

Another possible factor is changing societal perspectives on hormonal contraceptives, which may have introduced a cohort effect. In recent years, negative (social) media coverage and increasing scepticism about hormonal contraceptives have led to increased hesitancy among women considering their use.^6,9,31^ This shift could contribute to a ‘healthy user bias’ wherein adolescents who view themselves as mentally resilient are more likely to opt for OCP; this could have skewed the association towards fewer depressive symptoms among OCP users.^9,31^ Despite adjusting for previous psychopathology to reduce healthy user bias, our analysis indicates fewer depressive symptoms in OCP users. However, as previous psychopathology was measured on average 2 years earlier, residual healthy user bias could not be entirely excluded.

### Strengths and limitations

This study had numerous strengths, including its study population of young individuals that were likely to be first-time users of OCP. This resulted in a relatively homogeneous population, in which, for most participants, the choice of contraceptives was not influenced by earlier experiences. Another strength of this study was its inclusion of information on important sociodemographic variables, including ethnic background, household income, educational level and sexual activity, to adjust for potential confounding. In particular, adjustment for previous psychopathology was a critical aspect facilitated by the longitudinal nature of the current study.

There were also some limitations. First, observational data were used; therefore, causal inference could not be established. Second, we did not take into account the duration of OCP use within the past 2 years and thus could not conclude anything about differences in duration of use. However, considering the low number of individuals who discontinued OCP use, it is likely that OCP use was relatively consistent, suggesting that variations in duration would not significantly affect our findings. Also, based on our procedure of collecting data on OCP use, misclassification could have occurred if adolescents indicated they were not sexually active but took contraceptive medication without the knowledge of the parent. Common factors are unlikely to drive both misclassification of OCP and reporting of the outcome; thus, in this case, the measurement error would be likely to bias associations towards the null.^32^

We also had insufficient data to explore the type of OCP used. However, based on Dutch guidelines for general practitioners, the first choice of OCP is a combination of levonorgestrel and ethinyl estradiol.^33^ As our study population consisted of first-time users, it is likely that most participants used this OCP type. It is also the most used subtype of oral hormonal contraceptives in The Netherlands.^34^ In addition, our knowledge regarding the menstrual cycles of the participating girls, both OCP users and non-users, was limited. Variations in the experience of menstrual cycles among adolescents, such as the presence or absence of monthly menstruations, could affect mood. This is especially relevant considering factors such as premenstrual syndrome and their effects on quality of life.^35^ Premenstrual disorders contribute to gender differences in mental health, particularly internalising problems, emerging during adolescence.^29^ However, it is important to note the complexity of this issue, as some girls may initiate OCP use precisely to manage cycle-related mood problems.^35^ Moreover, although the possibility of a healthy survivor bias arises from girls discontinuing OCP use owing to adverse mood effects, the rarity of such discontinuations in our study population mitigates this concern.

### Implications and future directions

This study demonstrated a notable association between use of OCP and fewer internalising problems in adolescent girls compared with their non-using counterparts, with the strongest association observed in girls with clinical-level pre-existing internalising problems. This association persisted after adjustment for numerous confounders including age, multiple socioeconomic factors, prior internalising problems, previous OCP use and sexual activity. Although our results stem from an observational study, limiting our ability to draw causal conclusions, and healthy user bias may partially explain the findings, they suggest that OCP use may not increase the risk of depressive symptoms in adolescent girls and could in fact provide benefit to those with pre-existing internalising problems. Future research should concentrate on the various types and durations of OCP use, as well as the motivations behind starting or discontinuing their use. Attention to menstruation-related symptoms, such as polycystic ovary syndrome and premenstrual syndrome, is essential to better understand the multifaceted impacts of OCPs. Adolescent girls could significantly benefit from accurate and supportive counselling provided by healthcare professionals, taking individual factors such as mental health history in consideration and aiding them in making well-informed choices regarding contraceptive use.

## Data Availability

Data materials and code that support the findings of this study are available upon request from the principal investigator of the iBerry study: N.H.G.-v.M. (n.grootendorst@erasmusmc.nl).

## References

[1] Whiteford HA , Degenhardt L , Rehm J , Baxter AJ , Ferrari AJ , Erskine HE , et al. Global burden of disease attributable to mental and substance use disorders: findings from the Global Burden of Disease Study 2010. Lancet 2013; 382: 1575–86. 23993280 10.1016/S0140-6736(13)61611-6

[2] Noble RE. Depression in women. Metabolism 2005; 54(Suppl 1): 49–52. 10.1016/j.metabol.2005.01.014 15877314

[3] Kuehner C. Why is depression more common among women than among men? Lancet Psychiatry 2017; 4: 146–58. 27856392 10.1016/S2215-0366(16)30263-2

[4] Douma SL , Husband C , O’Donnell ME , Barwin BN , Woodend AK. Estrogen-related mood disorders: reproductive life cycle factors. ANS Adv Nurs Sci 2005; 28: 364–75. 16292022 10.1097/00012272-200510000-00008

[5] Frokjaer VG. Pharmacological sex hormone manipulation as a risk model for depression. J Neurosci Res 2020; 98: 1283–92. 32399989 10.1002/jnr.24632 PMC7383584

[6] Le Guen M , Schantz C , Regnier-Loilier A , de La Rochebrochard E. Reasons for rejecting hormonal contraception in Western countries: a systematic review. Soc Sci Med 2021; 284: 114247. 34339927 10.1016/j.socscimed.2021.114247

[7] United Nations, Department of Economic and Social Affairs, Population Division. Contraceptive Use by Method 2019: Data Booklet (ST/ESA/SER.A/435). UN, 2019.

[8] Rutgers, National Institute for Public Health and the Environment (RIVM), Statistics Netherlands (CBS). Monitor Seksuele Gezondheid 2023: Anticonceptiegebruik in Nederland. [Sexual Health Monitor 2023: Contraceptive Use in the Netherlands.] Rutgers, 2023.

[9] Martell S , Marini C , Kondas CA , Deutch AB. Psychological side effects of hormonal contraception: a disconnect between patients and providers. Contracept Reprod Med 2023; 8: 9. 36647102 10.1186/s40834-022-00204-w PMC9842494

[10] Skovlund CW , Morch LS , Kessing LV , Lidegaard O. Association of hormonal contraception with depression. JAMA Psychiatry 2016; 73: 1154–62. 27680324 10.1001/jamapsychiatry.2016.2387

[11] Johansson T , Vinther Larsen S , Bui M , Ek WE , Karlsson T , Johansson A. Population-based cohort study of oral contraceptive use and risk of depression. Epidemiol Psychiatr Sci 2023; 32: e39. 37303201 10.1017/S2045796023000525 PMC10294242

[12] de Wit AE , Booij SH , Giltay EJ , Joffe H , Schoevers RA , Oldehinkel AJ. Association of use of oral contraceptives with depressive symptoms among adolescents and young women. JAMA Psychiatry 2020; 77: 52–9. 31577333 10.1001/jamapsychiatry.2019.2838 PMC6777223

[13] Robakis T , Williams KE , Nutkiewicz L , Rasgon NL. Hormonal contraceptives and mood: review of the literature and implications for future research. Curr Psychiatry Rep 2019; 21: 57. 31172309 10.1007/s11920-019-1034-z

[14] McKetta S , Keyes KM. Oral contraceptive use and depression among adolescents. Ann Epidemiol 2019; 29: 46–51. 30674431 10.1016/j.annepidem.2018.10.002 PMC6349422

[15] Lundin C , Wikman A , Lampa E , Bixo M , Gemzell-Danielsson K , Wikman P , et al. There is no association between combined oral hormonal contraceptives and depression: a Swedish register-based cohort study. BJOG 2022; 129: 917–25. 34837324 10.1111/1471-0528.17028

[16] de Wit AE , de Vries YA , de Boer MK , Scheper C , Fokkema AA , Schoevers RA , et al. Hormonal contraceptive use and depressive symptoms: systematic review and network meta-analysis of randomised trials. BJPsych Open 2021; 7: e110. 34099098 10.1192/bjo.2021.64 PMC8220855

[17] Toffol E , Heikinheimo O , Koponen P , Luoto R , Partonen T. Further evidence for lack of negative associations between hormonal contraception and mental health. Contraception 2012; 86: 470–80. 22465115 10.1016/j.contraception.2012.02.014

[18] Keyes KM , Cheslack-Postava K , Westhoff C , Heim CM , Haloossim M , Walsh K , et al. Association of hormonal contraceptive use with reduced levels of depressive symptoms: a national study of sexually active women in the United States. Am J Epidemiol 2013; 178: 1378–88. 24043440 10.1093/aje/kwt188 PMC3888252

[19] Grootendorst-van Mil NH , Bouter DC , Hoogendijk WJG , van Jaarsveld S , Tiemeier H , Mulder CL , et al. The iBerry study: a longitudinal cohort study of adolescents at high risk of psychopathology. Eur J Epidemiol 2021; 36: 453–64. 33796978 10.1007/s10654-021-00740-w PMC8076148

[20] Bouter D , Ravenbergen S , de Neve-Enthoven N , Zarchev M , Mulder C , Hoogendijk W , et al. Five-year follow-up of the iBerry Study: screening in early adolescence to identify those at risk of psychopathology in emerging adulthood. Eur Child Adolesc Psychiatry 2024; 33: 4285–94. 38772966 10.1007/s00787-024-02462-2 PMC11618212

[21] Muris P , Meesters C , van den Berg F. The Strengths and Difficulties Questionnaire (SDQ). Further evidence for its reliability and validity in a community sample of Dutch children and adolescents. Eur Child Adolesc Psychiatry 2003; 12: 1–8. 10.1007/s00787-003-0298-2 12601558

[22] Bouwmans CAM , Schawo SJ , Jansen DEMC , Vermeulen K , Reijneveld M , Hakkaart-van Roijen L. Handleiding Vragenlijst Intensieve Jeugdzorg: Zorggebruik en productieverlies [Manual for the Intensive Youth Care Questionnaire: Health Care Utilization and Productivity Loss]. Erasmus MC, 2012.

[23] Sheppard R , Deane FP , Ciarrochi J. Unmet need for professional mental health care among adolescents with high psychological distress. Aust N Z J Psychiatry 2018; 52: 59–67. 28486819 10.1177/0004867417707818

[24] Rasic D , Hajek T , Alda M , Uher R. Risk of mental illness in offspring of parents with schizophrenia, bipolar disorder, and major depressive disorder: a meta-analysis of family high-risk studies. Schizophr Bull 2014; 40: 28–38. 23960245 10.1093/schbul/sbt114 PMC3885302

[25] Tellegen PJLJ. SON-R 6-40 Manual: Justification, Instructions, Norms. Hogrefe, 2011.

[26] R Core Team. R: A Language and Environment for Statistical Computing. R Foundation for Statistical Computing, 2021 (https://www.r-project.org/).

[27] Van Buuren SG-OK. mice: multivariate imputation by chained equations in R. J Stat Softw 2011; 45: 1–67.

[28] Jahanfar S , Mortazavi J , Lapidow A , Cu C , Al Abosy J , Morris K , et al. Assessing the impact of contraceptive use on mental health among women of reproductive age – a systematic review. BMC Pregnancy Childbirth 2024; 24: 396. 38816797 10.1186/s12884-024-06587-9 PMC11137968

[29] Li Y , Jiang J , Halldorsdottir T , Zhu H , Bertone-Johnson E , Valdimarsdottir UA , et al. Premenstrual disorders and gender differences in adolescent mental health. J Affect Disord 2023; 340: 930–7. 37543115 10.1016/j.jad.2023.08.009

[30] Lopez LM , Kaptein AA , Helmerhorst FM. Oral contraceptives containing drospirenone for premenstrual syndrome. Cochrane Database Syst Rev 2012; 2: CD006586. 10.1002/14651858.CD006586.pub4 22336820

[31] Hellstrom A , Gemzell Danielsson K , Kopp Kallner H. Trends in use and attitudes towards contraception in Sweden: results of a nationwide survey. Eur J Contracept Reprod Health Care 2019; 24: 154–60. 30920325 10.1080/13625187.2019.1581163

[32] VanderWeele TJ , Hernan MA . Results on differential and dependent measurement error of the exposure and the outcome using signed directed acyclic graphs. Am J Epidemiol 2012; 175: 1303–10. 22569106 10.1093/aje/kwr458 PMC3491975

[33] Barnhoorn PC , Bruinsma ACA , Bouma M , Damen Z , De Swart SM , Koetsier MJE , et al. NHG Guideline on Contraception. Dutch College of General Practitioners (NHG), 2020.

[34] Dutch Foundation for Pharmaceutical Statistics. Hormonal contraception: usage declines again. Pharmaceutical Weekly 2022; 157.

[35] Itriyeva K. Premenstrual syndrome and premenstrual dysphoric disorder in adolescents. Curr Probl Pediatr Adolesc Health Care 2022; 52: 101187. 35534402 10.1016/j.cppeds.2022.101187

